# An examination of risk factors that moderate the body dissatisfaction-eating pathology relationship among New Zealand adolescent girls

**DOI:** 10.1186/s40337-018-0225-z

**Published:** 2018-11-19

**Authors:** Juliet K. Rosewall, David H. Gleaves, Janet D. Latner

**Affiliations:** 1 Department of Psychology, University of Canterbury, Christchurch, New Zealand; 2 Child and Adolescent Eating Disorder Service, South London and Maudsley NHS Foundation Trust, London, UK; 3 School of Psychology Social Work and Social Policy, University of South Australia, Adelaide, Australia; 4 Department of Psychology, University of Hawaii, Honolulu, HI USA

**Keywords:** Adolescent eating pathology, Body dissatisfaction, Moderators, Risk factors

## Abstract

**Background:**

Eating disorders (EDs) and their subclinical variants are important health concerns for adolescent girls, and body dissatisfaction is a more common yet often debilitating experience that typically precedes the development of an ED. Despite this fact, little is known about what makes girls who are dissatisfied with their bodies more likely to engage in pathological eating behaviors. The present study explored eating pathology among a sample of adolescent girls from New Zealand and examined a variety of established risk factors that may moderate the relationship between body dissatisfaction (BD) and eating pathology.

**Methods:**

Adolescent girls aged between 14 and 18 (*N* = 231) completed questionnaires assessing eating pathology, BD, negative affect, perfectionism, self-esteem, teasing and sociocultural pressure. Regression analyses tested for moderator effects to examine which variables moderated the relationship between BD and eating pathology.

**Results:**

The analyses indicated that high levels of socially prescribed and self-oriented perfectionism, negative affect, perceived pressure from the media, and low levels of self-esteem all strengthened the relationship between BD and eating pathology.

**Conclusions:**

The results highlight potential factors that may make adolescent girls who are dissatisfied with their bodies more susceptible to eating pathology.

## Plain English summary

Many adolescent girls report being dissatisfied with their shape and weight. Body dissatisfaction can lead to negative outcomes, including eating pathology or unhealthy attitudes and behaviors towards food and eating. Eating pathology can be dangerous for adolescent girls given both the psychological and physical effects on a growing young person. However, not all adolescents who are dissatisfied with their bodies have unhealthy attitudes and behaviors towards food. This study looked at different factors that may increase the relationship between body dissatisfaction and eating pathology, or make it stronger. In other words, we wanted to find out what factors made someone who was dissatisfied more likely to also have unhealthy eating attitudes and behaviors. We gave 231 adolescent girls questionnaires measuring eating pathology, body dissatisfaction, mood, perfectionism, self-esteem, teasing and pressure to lose weight from the media and from others. We found that adolescent girls who were dissatisfied with their bodies, and who reported high levels of perfectionism, low mood, felt pressured by the media to lose weight or had low self-esteem reported greater levels of eating pathology. This is important to know as potentially working to reduce these factors among adolescents could protect those who are dissatisfied with their bodies from developing eating pathology.

## Background

Eating disorders (EDs) are important health concerns among young people due to their longstanding psychological and physical effects [1]. EDs are typically preceded by significant body dissatisfaction and the use of weight control strategies, such as restrictive dieting, which increase in severity and frequency over time [2]. Indeed, subclinical variants of EDs are also detrimental to health and wellbeing, affect a greater number of individuals, and could develop into a diagnosable ED [3]. Because of the complexities involved in treating an ED, particularly anorexia nervosa (AN), research focusing on risk factors that contribute to the development of pathological eating attitudes and behaviors is essential. Prevention efforts may be most effective when they focus on reducing established risk factors of ED development among adolescents; however, these are limited by our lack of understanding of these risk factors [4].

The sociocultural model of eating pathology purports that body dissatisfaction, and subsequent eating pathology, is/are associated with the pressure to be thin from one’s social environment [5] by reinforcing the message that thinness leads to social rewards such as acceptance and happiness [6]. According to this model, those who internalize the media-espoused thin ideal may be more likely to perceive a discrepancy between their appearance and the thin ideal. This internalization leads to body dissatisfaction and efforts to lose weight in order to decrease the discrepancy and attain the thin ideal. Evidence for the sociocultural model has been demonstrated in adolescent populations [7].

Among adolescents, body dissatisfaction (BD) is a common occurrence and, as the sociocultural model suggests, one of the most robust predictors of eating pathology [6, 8–12]. In a New Zealand study, Fear, Bulik and Sullivan [13] reported that 71% of adolescent girls experienced significant BD. Similarly, a New Zealand national wellness survey conducted by Wood and colleagues [14], found that 39% of adolescent girls were happy with their body, suggesting that the remaining 61% were not. Longitudinally, Neumark-Sztainer, Paxton, Hannan, Haines, and Story [15] found a strong and consistent relationship between BD and unhealthy weight control behaviors over a five-year period, even after controlling for BMI. Similarly, in their prospective study of adolescent girls, Rohde, Stice and Marti [16] reported that, of all the predictors they measured, BD was the most consistent predictor of future eating pathology, was significant at the four adolescent timepoints they measured (ages 13, 14, 15 and 16) and increased the likelihood of developing an eating disorder by 68%.

Given its “normative” nature [17], BD does not always result in an ED. Indeed, a study comparing adolescent girls with either AN, bulimia nervosa (BN), subthreshold AN, subthreshold BN or no ED found that BD was a common concern for each of these groups [18]. Although BD is common, EDs are relatively rare, and risk factors are likely to interact with each other [19]. Researchers have tried to explore the factors that may interact with BD in predicting eating pathology.

Using solely adult samples, moderator studies have examined various factors that affect the relationship between BD and eating pathology. In their longitudinal study, Vohs, Bardone, Joiner, Abramson, and Heatherton [20] found a three-way interaction effect between perfectionism, BD and self-esteem among 18 to 20 year old women. Perfectionistic women with high self-esteem tended to engage in adaptive weight control behaviors, whereas perfectionistic women with low self-esteem tended to engage in more maladaptive behaviors to control their weight. However, subsequent research has not always found support for this model [21]. In their study of adolescent girls followed prospectively over 1 year, Shaw, Stice, and Springer [22] also did not find this interaction effect to be statistically significant. Looking at perfectionism alone, in their study of college women, Downey and Chang [23] found that socially prescribed perfectionism, characterized by perceived high expectations from others, fear of negative evaluation and avoidance of the disapproval of others [24], moderated the pathway between BD and bulimic behaviors, and dieting. Brannan and Petrie [25] found similar results in their college sample with socially prescribed perfectionism moderating the effects of BD on bulimic symptoms, and self-oriented perfectionism, characterized by the setting of high personal standards and the scrupulous evaluation of one’s own behavior [26], serving as a moderator for anorexic symptoms only. In their longitudinal study of adolescent girls, Tyrka and colleagues [27] found that perfectionism at adolescence was a significant predictor of the onset of AN in young adulthood. The authors posited that girls with perfectionistic standards might shy away from the demands of adolescence, a time when flexibility is necessary, and devote their efforts to controlling their weight. In a subsequent longitudinal study with adolescents, Boone, Soenens, and Luyten [28] found that BD moderated the link between perfectionism and drive for thinness and overvaluation of shape and weight, but not bulimic symptoms. However, that study used the Frost Multidimensional Perfectionism Scale [29], an adult measure, which does not specifically measure self-oriented or socially prescribed perfectionism. These studies identified the moderating effect of perfectionism on the BD to eating pathology pathway, potentially due to having unrealistic expectations of shape and weight and increased inclination to strive for the thin ideal through extreme and pathological eating behaviors.

Other risk factors have also been **s**hown to interact with BD to increase the likelihood of eating pathology among adults. Depression and anxiety have been found to moderate the pathway between BD and eating pathology among adult women [30]. Using an adolescent sample, Rodgers, Paxton, and Chabrol [31] found that depression moderated the impact of sociocultural influences on eating concerns cross-sectionally; however, the researchers did not specifically study the relationship between BD and eating pathology. Leon and colleagues [32] found that, of the factors they studied, negative affect was the only significant predictor of disordered eating over a 3 to 4-year period during adolescence. This finding was further replicated in an independent sample of girls. It is possible that an adolescent girl could engage in pathological eating behaviors to help reduce negative mood states related to body dissatisfaction.

A longitudinal study by Button and colleagues [33] found that low self-esteem at an early age was predictive of later eating pathology in adolescent girls. Not only has low self-esteem been postulated as a risk factor, the Vohs et al. [20] study suggests that low self-esteem is a key moderating factor in the development of bulimic symptomatology. Moreover, Twamley and Davis [34] found that high self-esteem weakened the relationship between BD and eating pathology in women, meaning that these women were less likely to engage in unhealthy behaviors despite being dissatisfied. It is therefore plausible that adolescent girls with low levels of self-esteem may also be less likely to identify and focus on other personal strengths, making them more susceptible to the effects of BD [34].

Although we are not aware of any studies of moderator effects that have tested the interaction between BD and weight-related teasing, weight -related teasing has been found to predict eating pathology. In a large prospective study, Haines and colleagues [35] reported that adolescent girls who were teased were more likely than their peers to become dieters. The effects of teasing upon eating pathology have been shown to occur well into adulthood [36]. Another longitudinal study of general teasing in adolescence found that victims of teasing were at increased risk for symptoms of AN and BN [37]. These relationships occurred after controlling for prior ED symptoms, psychiatric status and family difficulties; however, in this study, the relationships did not extend into young adulthood. Similarly, perceived pressure to lose weight from others (particularly, family members and friends) is related to increased levels of eating pathology in adolescent girls. In a cross-sectional study of adolescent girls’ experiences of parental pressure, both talking about weight at home and maternal dieting was associated with increased eating pathology [38]. In addition to parental pressure, Paxton and colleagues [39] described how adolescent girls in the same friendship group share similar levels of dietary restraint, weight concerns, and extreme weight control practices. Pressure from peers, or peer competition, has been shown to predict eating pathology, both concurrently and prospectively, over and above that of social media and television influences [40]. Although weight-related teasing and pressure to lose weight are directly related to eating pathology, moderator analyses could reveal whether these factors increase eating pathology among those already dissatisfied with their bodies.

Research with adolescents that explores these factors is needed to determine why some young people who are dissatisfied develop eating pathology, why some do not develop eating pathology, and what factors may lessen risk**.** Such research may guide prevention efforts that aim to mitigate the effects of BD on eating pathology. Prevention is especially important during adolescence given that EDs tend to develop during this time, individuals who develop eating pathology during adolescence are at higher risk for eating pathology 10 years later [41] and once an eating pathology has developed into a full-blown ED, treatment can be challenging [42].

Guided by the sociocultural model of eating pathology, the current study explored the impact of the above-mentioned factors in an adolescent sample, focusing on the well-established relationship between BD and eating pathology. Given that weight-related teasing and social pressure to be thin are both prevalent in adolescence and related to increased BD and eating pathology [35, 43], we tested whether these factors could also act as moderators. Although the sociocultural model posits that these factors precede BD, higher levels of these factors might still strengthen the BD to eating pathology pathway. For example, among girls who are dissatisfied with their bodies, the media or others may erroneously instruct them on how to achieve the thin ideal, through restrictive dieting and overexercising [44], or such girls may model the pathological eating behaviors they see in the media or among friends. We predicted that high perfectionism, negative affect, teasing, perceived pressure to be thin, and low self-esteem would each strengthen the relationship between BD and eating pathology.

## Methods

### Participants and procedures

Participants were 231 adolescent girls aged between 14 and 18 years, recruited from three state high schools throughout Christchurch, New Zealand. The schools’ decile ratings, a government-issued score on a 10-point scale indicating socio-economic level [45], ranged from 2 to 9, suggesting that both high and low socio-economic communities were represented in the sample.

Eight secondary schools were invited to take part in this study, and three agreed to participate. Once a school agreed to participate, letters were sent home to recruit female participants. Informed consent was obtained from each participant and their parent or guardian. Questionnaires were group-administered and completed during class time. One school did not consent to their students being measured for height and weight; therefore, these measurements were based on participant self-report. For the remainder of the participants, a research assistant conducted height and weight measurements in a separate room.

### Measures

#### Demographic information

A questionnaire assessed age, ethnicity, and mother and father/guardian occupation.

#### Socio-economic status

The New Zealand Socio-Economic Index (NZSEI) [46] is a widely used occupationally- based measure of socio-economic status (SES) used in this study. Participants completed details about their parent/ guardian’s occupation in order to derive a socio-economic index score.

#### Eating pathology

We used the Eating Attitudes Test − 26 (EAT-26) [47] to measure eating pathology. The EAT-26 is a widely used 26-item self-report questionnaire that measures eating disturbances, originally designed to measure the symptoms of AN. An example item is “I feel terrified about being overweight”. The EAT-26 is a shortened version based on the factor analysis of the EAT-40 and correlates highly with the original measure (*r* = .98). The measure uses a 6-point scale ranging from “always” to “never” and the most symptomatic response is given a score of three, the second most symptomatic response is assigned a score of two and the third, one. The other three less symptomatic responses are given a score of zero. Thus, scores range from 0 to 78. Although the EAT-26 does not yield a specific diagnosis of an ED, it can be an efficient screening tool in which a score at or above a cut-off score of 20 is indicative of eating pathology [48]. The EAT-26 has demonstrated good score reliability with adolescents [40]. In the current study, Cronbach’s alpha was .90.

#### Body dissatisfaction

The Stunkard Body Figure Drawings [49] is a measure of body image perception and assesses BD in adolescents and adults. Participants were presented with nine figures ranging in size from very thin to obese, and were required to identify the figure that represents their perceived current body size and the figure that represents their ideal body size. A discrepancy score is obtained by subtracting ideal body size from perceived current size. Fair to very good test-retest reliability has been reported for scores on this measure [50].

The Eating Disorders Inventory-Body Dissatisfaction subscale (EDI-BD) [51] is a nine-item measure of BD that asks participants to rate statements about dissatisfaction with parts of their body (e.g. “I think my thighs are too large”) on 6-point scale ranging from “always” to “never”. Total scores range from zero to a possible score of 27. The most symptomatic response is given a score of three, and the second and third most symptomatic responses are assigned scores of two and one, respectively. The other three least symptomatic responses are given a score of zero. This subscale has demonstrated good score reliability and validity in adolescent populations [52, 53]. In the current study, Cronbach’s alpha was .83.

#### Perfectionism

The Child and Adolescent Perfectionism Scale (CAPS) [54] is a 22-item multidimensional perfectionism scale used to assess socially prescribed (SP) perfectionism (e.g. “My family expects me to be perfect”) and self-oriented (SO) perfectionism (e.g. “I get mad at myself when I make a mistake”) in children and adolescents. The CAPS consists of 5-point Likert scales and higher scores on the scale indicate greater degrees of perfectionism. SO perfectionism scores range from 12 to 60 and SP perfectionism score range from 10 to 50. The CAPS has demonstrated good score reliability [55]. In the current study, the Cronbach’s alpha for the SP perfectionism scale was .82, and for SO perfectionism was .75.

#### Self-esteem

The Rosenberg Self-esteem Scale (RSES) [56] is a widely used 10-item measure of general self-esteem. An example item is “I feel I do not have much to be proud of”. The RSES consists of a four-point Likert scale, ranging from “strongly agree” to “strongly disagree”. Scores range from 10 to 40, with high scores indicating low self-esteem and low scores, high self-esteem. The RSES has demonstrated good reliability in previous research [57]. In the current study, Cronbach’s alpha was .86.

#### Perceived pressure to lose weight

The Perceived Pressure to Lose Weight subscale of the Sociocultural Influences on Body Image and Body Change Questionnaire [58] is an 18-item measure assessing (1) perceived pressure from others (father, mother, best female friend and best male friend) and (2) perceived pressure from the media to lose weight. Example items are e.g., “Does your best female friend diet to lose weight?” and “Do the media give you the idea that you should eat less to lose weight?” This self-report measure requires the participants to rate each item on a 5-point scale ranging from “never” to “always”. Higher scores are indicative of higher perceived pressure to lose weight. In the current study, Cronbach’s alpha was .73 for pressure to lose weight from others and .82 from the media, respectively.

#### Negative affect

The Positive and Negative Affect Scale (PANAS) [59] is a measure of positive and negative affect. We only used the 10-item negative affect scale in this study. Participants were required to rate the degree to which different words (e.g., “scared”) describe how they have felt during the past few weeks, on a scale of 1 (very slightly or not at all) to 5 (extremely). PANAS scores range from 10 to 50. Good reliability and validity data for young people have been reported for the PANAS [60–62]. Cronbach’s alpha in this study was .87.

#### Weight-related teasing

The Perception of Teasing Scale (POTS) [63] is an 11-item measure of teasing and its effect. This study only examined the weight-related teasing subscale. An example item is “People made fun of you because you were heavy”. Participants were required to rate teasing frequency on a Likert scale of “never” to “very often”. High scores on this scale are indicative of a greater frequency of negative verbal commentary. Potential scores range from 6 to 30. The POTS has reliably assessed teasing in adolescents and children [64]. In the current study, Cronbach’s alpha was .88.

### Statistical analyses

Data analyses were performed using SPSS version 24 with PROCESS for SPSS 2.16.3 [65]. Descriptive statistics were calculated to determine the sample composition. Regression analyses tested for moderator effects to examine which variables moderated the relationship between BD and eating pathology. Other than when we tested three-way interactions, the two-way interactions were tested one at a time. A composite BD score of the EDI-BD and Stunkard Figure Drawings was computed using principal components analysis and was subsequently used in the regression model. This served as a cautionary measure to reduce multicollinearity and because these two variables were measuring two aspects of BD [66, 67]. A subsequent series of hierarchical multiple regressions was computed to test for moderator effects, with eating pathology as the dependent variable. Within PROCESS, predictor and moderator variables were centered prior to testing interaction effects. All lower-order effects were included in the models. Thus, for tests of three-way interactions, all 3 two-way interactions were also included in the model. BMI and age were also included as covariates. To observe the nature of each statistically significant interaction, conditional effects at high, medium and low values of the moderator variables were tested. High, medium and low were tests of effects at one SD above the mean, the mean, and one SD below the mean respectively [68].

## Results

### Sample characteristics

The mean age of the sample was 15.5 years (*SD* = 1.05). The percentages of the girls who reported being 14, 15, 16, 17, or 18 were 16.5%, 37.2%, 27.3%, 15.6% and 3.5%, respectively. The mean body mass index (BMI; kg/m^2^) was 21.9 (*SD* = 3.87); 67% of the girls had a BMI in the normal range (5th to <85th percentile), 16% fell in the underweight range (< 5th percentile), 12% in at risk for overweight range (85th to 95th percentile), and 5% in the overweight range (95th percentile and above) [69]. The ethnic composition of the sample was 73.7% New Zealand European, 10.3% New Zealand Māori, 5.6% Asian, 2.6% Pacific Island and 3% Other. Ethnic distribution was similar to the population statistics of children and youth in Christchurch city [70]. The mean SES score was 3.8, which falls in the middle-SES range [46].

In terms of missing data, a small number of participants left all items of the POTS and media pressure scale blank (15 and 5, respectively) and were excluded from the analyses. Of the remaining participants, 62 participants were missing at least one item on any measure with the majority missing only one item. To deal with remaining missing data, we used the expectation-maximization (EM) method of data imputation (see [71]). Due to incomplete data, the majority of analyses were conducted with 207 participants except the analyses including the POTS, which were conducted with 195.

Mean scores and intercorrelations are shown in Table 1. Although individuals generally reported low levels of eating pathology on the EAT-26, a total of 28 participants (12.1%) scored above the recommended cut-off of 20 on the EAT-26, indicative of eating pathology [24]. Thirty-seven participants (15.9%) reported having engaged in compensatory behavior, such as vomiting or laxatives, during the past 6 months.

**Table 1:** Descriptive Statistics, Zero-order Correlations Between Variables of Interest and Cronbach’s Alphas for each Measure

*α*	1	2	3	4	5	6	7	8	9	10	11
1 Eating pathology	.*90*	.24**	.50**	.50**	−.36**	.36**	.32**	.45**	.23**	−.09	.30**
2 BMI		*N/A*	.55**	.50**	−.25**	.13	.07	.19**	.49**	−.05	.28**
3 Figure drawings			*N/A*	.70**	−.31**	.24**	.16*	.31**	.37**	−.06	.31**
4 Body dissatisfaction				.*83*	−.51**	.24**	.15*	.33**	.35**	−.03	.35**
5 Self esteem					.*86*	−.30**	−.07	−.41**	−.35**	−.02	−.15*
6 SP perfectionism						.*82*	.60**	.34**	.10	−.15*	.24**
7 SO perfectionism							.*75*	.23**	.10	−.04	.16
8 Negative affect								.*87*	.15*	−.03	.20**
9 Weight teasing									.*88*	−.01	.34**
10 Media pressure										−.*82*	−.04
11 Perceived pressure											.*73*
*M*	9.76	21.97	1.16	10.88	28.74	28.49	35.33	21.67	8.29	9.96	16.32
*SD*	11.19	3.59	1.31	7.64	5.02	7.39	6.73	6.82	4.09	3.06	4.08

*n* = 195 (using listwise deletion); *p < .05. **p < .01; *SD* standard deviation, *SP perfectionism* socially prescribed perfectionism, *SO perfectionism* self-oriented perfectionism

### Moderator analyses

Regression coefficients for each moderation analysis are depicted in Table 2 and their level of significance and conditional effects are presented in Table 3.

**Table 2:** Summary from Regression Analysis of Individual Moderator Effects on the BD to Eating Pathology Pathway

Predictor	B	SE B	ΔR^2^	*t*	*p*
SO perfectionism x BD	.41	.09	.08**	4.69	<.001
SP perfectionism x BD	.37	.70	.06**	3.78	<.001
Self esteem x BD	−.33	.16	.02**	−2.03	.05
Perceived pressure x BD	.32	.26	.01	1.21	.22
Negative affect x BD	.33	.12	.05**	2.71	<.01
Media pressure x BD	.79	.28	.05**	2.79	<.01
Weight teasing x BD	.18	.17	.01	1.03	.30
SO perfectionism x self esteem x BD	.01	.03	.00	.27	.79
SP perfectionism x self esteem x BD	−.01	.02	.00	−.29	.77

*p < .05. **p < .01; BMI and age were added as covariates in each model and had no significant effect on interaction effects, *n* = 207, with the exception of the analysis using the POTS (weight-related teasing) where *n* = 195

**Table 3:** Conditional Effects of BD on Eating Pathology at High, Medium and Low Levels of each Moderator

Moderator	Level	Effect	se	*t*	*p*
SO perfectionism	High	8.22	1.17	7.06	<.001
	Med	5.87	1.13	5.21	<.001
	Low	3.51	1.37	2.59	.01
SP perfectionism	High	7.05	1.21	5.85	<.001
	Med	4.34	0.90	4.80	<.001
	Low	1.62	1.10	1.47	.14
Self esteem	High	3.49	1.83	1.90	.06
	Med	5.12	1.55	3.30	<.01
	Low	6.76	1.66	4.08	<.001
Negative affect	High	7.01	1.50	4.68	<.001
	Med	4.79	1.16	4.12	<.001
	Low	2.58	1.34	1.92	.06
Media pressure	High	8.23	1.49	5.53	<.001
	Med	5.78	1.13	5.13	<.001
	Low	3.34	1.37	2.44	.02

High, medium and low values are one SD above the mean, the mean and one SD below the mean, respectively

The interactions between both SP perfectionism and BD, and SO perfectionism and BD, as predictors of eating pathology, were statistically significant. As seen in the conditional effect values, among adolescent girls who scored high (or one SD above the mean) on SP perfectionism, the relationship between BD and eating pathology was greater than for those at the mean or one SD below the mean. Similarly, conditional effect values demonstrated that for those scoring higher on SO perfectionism, the relationship between BD and eating pathology was also greater. The three-way interactions between BD, self-esteem and both SP and SO perfectionism, in predicting eating pathology, were not statistically significant.

The interaction between BD and self-esteem, as a predictor of eating pathology, was statistically significant. Conditional effect values showed the relationship between BD and eating pathology increased at lower levels of self-esteem.

The interaction between BD and negative affect, in predicting eating pathology, was also statistically significant. High levels of negative affect strengthened the effect of BD on eating pathology. Tests of conditional effects indicated that the relationship between BD and eating pathology was strongest at higher levels of negative affect.

The interaction between BD and pressure from the media to lose weight, in predicting eating pathology, was statistically significant. As can be seen in the conditional effects, as media pressure becomes greater, the relationship between BD and eating pathology becomes stronger. There was no significant interaction found between BD and weight-related teasing or perceived pressure from others, in predicting eating pathology.

## Discussion

The current study revealed that SP and SO perfectionism, self-esteem, negative affect and perceived pressure from the media each moderated the relationship between BD and eating pathology. Similar to adult samples [30], among adolescents who are dissatisfied with their bodies, high levels of negative affect may increase the effects on eating pathology. Although causation cannot be inferred, negative affect may exacerbate the challenges of adolescence, such as the sometimes-unwanted physical changes associated with puberty. Pathological eating behaviors may be a means of providing relief from negative mood states, particularly when they are related to one’s BD [72]. However, as these data are not longitudinal, it is also possible that eating pathology leads to negative affect via feelings of shame and guilt [73] or that some unmeasured variable is responsible for the effects that we found. Consistent with adult samples, high levels of both SP and SO perfectionism increased the impact of BD on eating pathology. This finding suggests that girls who are dissatisfied with their bodies may be more likely to engage in pathological eating behaviors if they are highly perfectionistic, whereas girls who are low in perfectionism may be less likely to act on their BD. Theoretically, low perfectionism could decrease the likelihood adolescent girls engage in eating pathology because personal standards may be more realistic and the drive to achieve the thin body ideal may be less intense. Similarly, high levels of perfectionism may perpetuate a drive for thinness [28] and an inability to lose weight may be met with more extreme and unrealistic weight loss strategies to achieve the thin ideal. Similar to the findings of Shaw and colleagues [22], the three-way interaction between perfectionism, self-esteem and BD found by Vohs et al. [20] was not replicated. Again, given the cross-sectional nature of these findings it could be possible that eating pathology increases perfectionism via weight loss and the consequent exacerbation of weight loss efforts.

Consistent with Twamley and Davis [34], low self-esteem increased the relationship between BD and eating pathology. Low self-esteem might increase risk through preventing high-BD girls from viewing themselves in a more positive light, or by diminishing other strengths [34] making them more focussed on or affected by their BD. In a similar vein, media pressure real or perceived, might exacerbate her established view about her body and encourage an adolescent girl to pursue weight loss. Dissatisfied girls might also seek out weight-related media messages, or might be even more sensitive to them. However, as directionality cannot be assumed, it could also be possible that eating pathology leads to an increase in media pressure to lose weight, or a sensitivity to these messages. Interestingly, teasing and perceived pressure did not significantly moderate the BD-eating pathology pathway. In light of previous research, weight-related teasing and pressure might be directly associated with increased BD as opposed to moderating the BD-eating pathology relationship [12, 74].

To our knowledge, this was the first study in the past 20 years to have examined adolescent eating pathology in New Zealand and the first to examine established risk factors for eating pathology among New Zealand adolescent girls. Rates of eating pathology in this study were similar to those found internationally (as measured by exceeding the EAT-26 cutoff) [75, 76]; however, given that the sample was not randomly selected, we cannot be certain that our sample was representative and thus directly comparable to other overseas estimates.

A number of methodological limitations of this study need to be considered. The cross-sectional nature of the data makes it unclear what direction of causality (if any) exists among the variables. Although we can be guided by theoretical understanding, longitudinal research would identify risk factors over time and partially clarify causal relationships. Moreover, as the multiple moderators were not entered into one analysis, we cannot be sure which moderators were statistically significant above and beyond the effects of other potential moderators. Testing all of the interactions at once would have created multicollinearity concerns that would have made the estimates unstable, and may have made tests of moderation problematically underpowered and increased the chance of type-2 errors. As mentioned, the sample was not randomly selected. Although effort was made to recruit participants from schools that covered a wide range of socioeconomic areas, not all schools consented to participate. Moreover, data on enrolment or participation rates were not collected which again affects the representation of the sample. Questionnaire data were collected by self-report. Despite being reassured confidentiality, participants may not have been completely open and honest or, in the instance of the NZSEI, may not have known. Including a clinical interview or parental report may have improved accuracy, but would have been difficult with a large, non-clinical sample. Although BD and eating pathology occurs in males, this study only recruited female participants. Exploring these same potential moderator relationships in adolescent boys is greatly needed. Moreover, perceived pressure to lose weight was a collapsed variable containing items measuring pressure from father, mother and friends; therefore, individual sources of pressure were not identified.

## Conclusions

Despite these limitations, the current study increases our understanding of the relationships between BD and the established risk factors that may potentially make adolescent girls more susceptible to eating pathology. Without intervention, pathological eating could develop into a diagnosable ED. Prevention strategies that seek to reduce BD might be more effective if they address ways to curtail the effects of these moderating factors also. For example, rather than just addressing BD, targeted prevention strategies for those with high levels of BD could focus on reducing perfectionism (both SP and SO) through addressing maladaptive perfectionist beliefs about shape, weight and one’s control over their eating. In addition, such interventions could possibly benefit from an emotion regulation component to address negative affect. Negative affect may be alleviated by teaching skills that allow an adolescent to manage unwanted mood states in an adaptive fashion [32]. Similarly, self-esteem could be targeted by encouraging and modelling self-worth based on other areas of an individual, such as inner qualities, values, and meaningful social relationships. Prevention efforts that have targeted self-esteem have shown good results at reducing BD and eating pathology among adolescent girls [3]. Finally, media messages that convey beauty and thinness leading to success and happiness could also be challenged as can the accuracy of images in the media. Prevention programmes often address these unhelpful media messages, and have indeed demonstrated promising outcomes with adolescents [77]. If corroborated by longitudinal and/or experimental research, accentuating these moderating factors could help improve our prevention strategies and, ultimately, interrupt the chain of events that lead to eating pathology.

## Abbreviations

AN: Anorexia nervosa
BD: Body dissatisfaction
BMI: Body mass index
BN: Bulimia nervosa
CAPS: Child and Adolescent Perfectionism Scale
EAT-26: Eating Attitudes Test-26
EDI-BD: Eating Disorders Inventory- Body Dissatisfaction
EDs: Eating disorders
NZSEI: New Zealand Socio-Economic Index
PANAS: Positive Affect and Negative Affect Scale
POTS: Perception of Teasing Scale
RSES: Rosenberg Self Esteem
SES: Socio-economic status
SO: Self-Oriented
SP: Socially Prescribed
